# Hybrid Capture II-based human papillomavirus detection, a sensitive test to detect in routine high-grade cervical lesions: a preliminary study on 1518 women

**DOI:** 10.1038/sj.bjc.6690523

**Published:** 1999-07

**Authors:** C Clavel, M Masure, J-P Bory, I Putaud, C Mangeonjean, M Lorenzato, R Gabriel, C Quereux, P Birembaut

**Affiliations:** Laboratoire Pol Bouin, CHU de Reims, 45 rue Cognacq-Jay, Reims, 51092, France; Department of Obstetrics and Gynecology, CHU de Reims, Reims, 51100, France

**Keywords:** human papillomavirus, cervical cancer, cytological screening

## Abstract

Hybrid Capture II (HC-II) is a commercial human papillomavirus (HPV) detection test designed to detect 18 HPV types divided into high-risk and low-risk groups. We have tested 1647 scrapes from 1518 unselected women attending routine cytological screening by this assay for the detection of histologically proven high-grade lesions. The reliability of this test was also evaluated on 117 fresh cone biopsy samples. HPV DNA has been detected in 400 scrapes (24.3%), 296 containing a high-risk HPV (18.0%). All the smears evocative of high-grade lesions were positive for high-risk HPV, and high-risk HPV were detected in all the 34 cases presenting a histologically proven high-grade lesion and in 68 (97.1%) of the 70 cone biopsy samples showing a high-grade lesion or an invasive carcinoma. Thus, the sensitivity was superior to the sensitivity of cytology (85.3%). Nevertheless, the quantitative approach provided by the HC-II assay for the assessment of the viral load could not clearly distinguish among cases with or without high-grade lesions. Thus this assay is recommended for the screening of high-grade lesions on a large scale, in association with classic cytology. © 1999 Cancer Research Campaign


*British Journal of Cancer* (1999) **80**(9), 1306–1311
© 1999 Cancer Research Campaign
Article no. bjoc.1999.0523

### Hybrid Capture II-based human papillomavirus detection, a sensitive test to detect in routine high-grade cervical lesions: a preliminary study on 1518 women

**C Clavel[1], M Masure[1], J-P Bory[2], I Putaud[1], C Mangeonjean[1], M Lorenzato[2], R Gabriel[2], C Quereux[2] and P Birembaut[1]**

[1]Laboratoire Pol Bouin, CHU de Reims, 45 rue Cognacq-Jay, 51092 Reims, France; [2]Department of Obstetrics and Gynecology, CHU de Reims, 51100 Reims, France

**Summary** Hybrid Capture II (HC-II) is a commercial human papillomavirus (HPV) detection test designed to detect 18 HPV types divided into high-risk and low-risk groups. We have tested 1647 scrapes from 1518 unselected women attending routine cytological screening by this assay for the detection of histologically proven high-grade lesions. The reliability of this test was also evaluated on 117 fresh cone biopsy samples. HPV DNA has been detected in 400 scrapes (24.3%), 296 containing a high-risk HPV (18.0%). All the smears evocative of high-grade lesions were positive for high-risk HPV, and high-risk HPV were detected in all the 34 cases presenting a histologically proven high-grade lesion and in 68 (97.1%) of the 70 cone biopsy samples showing a high-grade lesion or an invasive carcinoma. Thus, the sensitivity was superior to the sensitivity of cytology (85.3%). Nevertheless, the quantitative approach provided by the HC-II assay for the assessment of the viral load could not clearly distinguish among cases with or without high-grade lesions. Thus this assay is recommended for the screening of high-grade lesions on a large scale, in association with classic cytology.

**Keywords:** human papillomavirus; cervical cancer; cytological screening

Persistent infection with oncogenic human papillomavirus (HPV) types, of which type 16 is the most prevalent, represents a major risk factor for the development of cervical carcinoma (Lorincz et al, 1992; Zur Hausen, 1994). Between 90 and 100% of these lesions are positive for such high-risk HPV by polymerase chain reaction (PCR) (Van den Brule et al, 1991; Bosch et al, 1995). Then infections with high-risk HPV are associated with a relative risk of between 8 and 11 for the development of squamous intra-epithelial lesions (SIL) (Koutsky et al, 1992) and only low-grade SIL containing high-risk HPVs progress to high-grade disease (Gaarenstroom et al, 1994). Because of this, there is an increasing interest in using HPV DNA detection, either alone or in addition to the classic cytological examination, as a method for screening cervical carcinoma or for triaging women with a cytological diagnosis of atypical squamous cells of undetermined significance (ASCUS) in their cervical smears (Cole, 1993; Richart and Wright, 1993; Cox et al, 1995). Nevertheless, the low sensitivity of HPV detection has led some authors to consider that HPV screening does not appear to be of value for identifying women with abnormal smears who can be safely followed up with cytological study alone (Kaufman et al, 1997). Indeed, HPV DNA testing in cervical scrapes offers a potentially automatic and cheap diagnostic assay without the sampling problems and subjectivity of cytology, but such an approach needs a specific, sensitive, reliable and easy to perform method.

*Received 19 October 1998*
*Revised 12 January 1999*
*Accepted 27 January 1999*

*Correspondence to:* C Clavel

Three years ago, a commercial HPV detection test, Hybrid Capture-I (HC-I), was introduced (Farthing et al, 1995; Schiffman et al, 1995; Sun et al, 1995). HC-I was a non-radioactive, relatively rapid, liquid hybridization assay designed to detect 14 HPV types divided into high-risk (types 16, 18, 31, 33, 35, 45, 51, 52 and 56) and low-risk (types 6, 11, 42, 43 and 44) groups. This test has been proposed to be used routinely on large series of women to improve the sensitivity and negative predictive value of cytological screening for SIL (Sun et al, 1995). The first studies were particularly promising, showing a good agreement with PCR results, although the method was less sensitive, with a detection of high-risk HPV DNA in 83–93% in high-grade SIL. These papers, and the following studies of selected populations, concluded that screening for cervical intraepithelial neoplasias (CIN) grade 2/3 could be significantly improved by HPV testing with the HC-I assay. However, in our own experience on a series of 1028 women attending routine screening, the overall sensitivity in detecting high-grade SIL with HC-I was only 71.2% and its positive predictive value was 17.8% (Clavel et al, 1998*a*). Classic cytological screening remained the most sensitive tool (84%) to detect high-grade SIL with a positive predictive value of 91.3%. Thus, we concluded that the crude sensitivity of the HC-I limited its use for the screening of high-grade lesions on a large scale.

Since this study, a second generation of HC, Hybrid Capture-II (Digene) (HC-II) using microtiter plates instead of tubes and detecting four additional viral oncogenic types (types 39, 58, 59 and 68) has been commercialized. The preliminary studies gave more reliable results with a lower detection limit of 0.2–1 pg HPV DNA $ml^{-1}$ (Lorincz, 1996). The aim of the present work was to study on a large series of unselected women attending routine cytological screening, the utility and efficiency of the HPV detection by the HC-II test to detect histologically proven high-grade lesions as compared with classic cytological screening, and the reliability of this test on various well-established high-grade SIL treated by cone excision.

### MATERIALS AND METHODS

A total of 1518 women with a mean age of 37 years (range 15–72 years) were recruited for the study between August 1997 and December 1998. This population included women who underwent their triennial routine screeening, women with high risk of sexually transmitted diseases and women followed in a family planning clinic, in the Department of Obstetrics and Gynecology of the CHU of Reims. All women were informed of the aim of the study and gave their consent. At gynaecological examination, two samples were taken: first, a cytological smear with an Ayre's spatula, then one scrape for the HC-II test with a cytobrush (Medscan, Uppsala, Sweden). We collected 1647 cervical smears and scrapes. Moreover, to test the sensitivity and the specificity of the detection of HPV DNA and of the viral load evaluated by the HC-II assay, we performed a pilot study with the same approach on 117 fresh samples of cone biopsies following the discovery of a high-grade SIL on a previous biopsy, from patients with a mean age of 37.5 years (range 20–58 years).

Specimens for HPV DNA testing were suspended in 1 ml of ViraPap/Viratype transport medium (Digene, Silver Spring, MD, USA) and stored at –20°C until further processing.

Smears were classified according to the Bethesda System for reporting cervical or vaginal cytological diagnosis. In our protocol (Figure 1), all the women presenting cytological abnormalities evocative of cervical lesions were systematically recalled for colposcopy in the next few weeks, at an interval ranging from 14 to 150 days (mean 90 days) after entry examination. Punch biopsy specimens were taken from the areas colposcopically suspicious for CIN. If colposcopy was negative, the woman was recalled 6 months later for a new cytological control and HPV testing. All the women with smears within normal limits but presenting a HPV infection were also systematically recalled 6 months later for a new cytological examination and HPV testing followed by colposcopy if a lesion and/or a persistent HPV infection was detected. Punch biopsy specimens were taken from the areas colposcopically suspicious for CIN. The women with a second HPV test positive without any detectable lesions were recalled 6 months later for a third control with cytological examination and HPV testing, and the same indications as above for colposcopy and biopsy. In total, 114 women had at least two repeated HPV tests and cytology screens, at an interval ranging from 30 to 450 days (mean 170 days). A total of 165 women had colposcopy and 129 were biopsied. The cytotechnicians and pathologists involved were not informed about the results of the HPV testing. All biopsy specimens were examined by two pathologists.

HPV DNA detection was performed by the commercially available HC-II System (Digene). All scrapes were analysed for the presence of low-risk HPV types 6, 11, 42, 43 and 44, and high-risk HPV types 16, 18, 31, 33, 35, 39, 45, 51, 52, 56, 58, 59 and 68. This enzyme-linked immunosorbent assay is based on a sandwich hybridization followed by a non-radioactive alkaline phosphatase reaction with chemoluminescence in microplates (Lorincz, 1996). The positive threshold of this test is 0.2 pg $ml^{-1}$ (1000 copies of HPV per test).

**Table 1** Prevalence of HPV infections according to age (patients with double infections were included in high-risk HPV infection)

| Age | Women | High-risk | Low-risk | Total HPV |
|---|---|---|---|---|
| <20 | 85 | 17 (20.0%) | 5 (5.9%) | 22 (25.9%) |
| 21–30 | 411 | 103 (25.1%) | 25 (6.1%) | 128 (31.1%) |
| 31–40 | 484 | 71 (14.7%) | 24 (4.9%) | 95 (19.6%) |
| 41–50 | 369 | 42 (11.4%) | 23 (6.2%) | 65 (17.6%) |
| 51–60 | 128 | 14 (10.9%) | 8 (6.2%) | 22 (17.2%) |
| >60 | 41 | 6 (14.6%) | 0 | 6 (14.6%) |
| Total | 1518 | 253 (16.7%) | 85 (5.6%) | 338 (22.3%) |

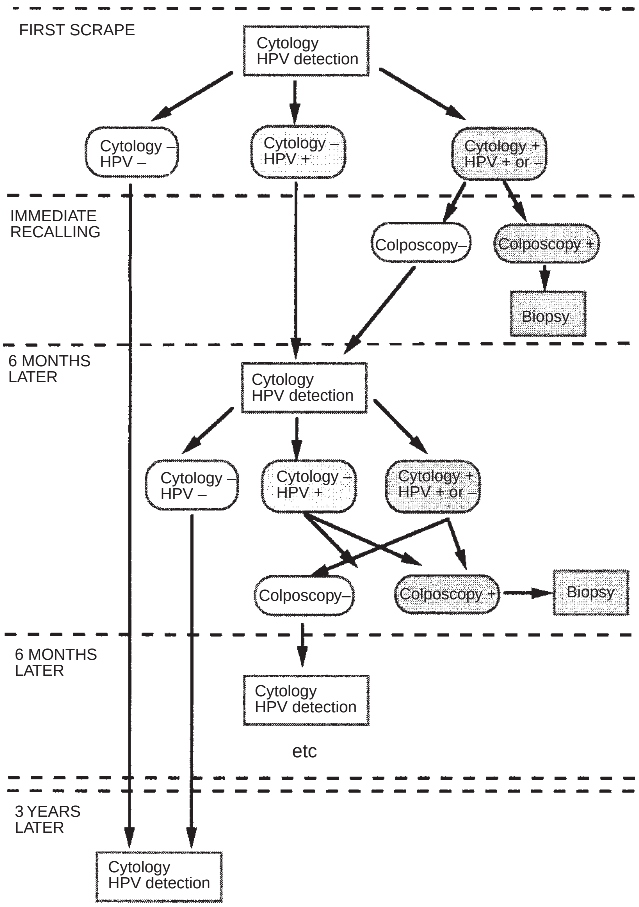

**Figure 1** Protocol for follow-up

Samples were classified as positive for HPV DNA if the relative light unit reading obtained from the luminometer was equal or greater than the mean of positive control values supplied by the HC-II kit. As some authors have reported that increasing HPV DNA levels of high-risk HPV types were the principal predictors of CIN (Cox et al, 1996), we used as proposed, the ratio relative light units per positive control values to quantify high-risk HPV DNA in our samples. Moreover, we added other positive controls such as SiHa cell lines (1–2 copies of HPV type 16 per cell) to check the reproducibility of the HC-II sensitivity.

**Table 2** Follow-up from the first smear to the final diagnosis

| Cytology | | | Histology | | |
|---|---|---|---|---|---|
| First smear | HPV | | Final histological diagnosis | HPV | |
| | HR | LR | | HR | LR |
| 1414 WL | 168 | 85 | 5 HG-SIL | 5 | 0 |
| | | | 7 LG-SIL | 7 | 0 |
| 23 ASCUS | 11 | 0 | 2 HG-SIL | 2 | 0 |
| | | | 5 LG-SIL | 5 | 0 |
| 56 LG | 43 | 4 | 2 HG-SIL | 2 | 0 |
| | | | 42 LG-SIL | 35 | 3 |
| 25 HG | 25 | 0 | 25 HG-SIL | 25 | 0 |

WL: without lesion; LG: low-grade lesion; HG: high-grade lesion; HR: high-risk HPV; LR: low-risk HPV.

**Table 3** Evaluation of cytology and high-risk HPV testing for the detection of a histologically proven high-grade lesion

| Methods | Sensitivity | Specificity | PPV |
|---|---|---|---|
| Cytology | 29/34 (85.3%) | 1409/1984 (94.9%) | 29/104 (27.9%) |
| HPV detection | 34/34 (100%) | 1265/1484 (85.2%) | 34/253 (13.4%) |
| High-risk HPV DNA level in conizations (ratio) | | | |
| >50 | 68.0% | 71.4% | 92.7% |
| >100 | 56.0% | 78.6% | 93.3% |
| >200 | 46.7% | 85.7% | 94.6% |
| >500 | 24.0% | 92.8% | 94.7% |

PPV: predictive positive value.

### RESULTS

Overall, 400 scrapes out of 1647 (24.3%) were positive for the detection of HPV by the HC-II test: 296 for high-risk HPV (18.0%) including 83 double infections and 104 low-risk HPV (6.3%). These positivities corresponded to 338 out of 1518 women (22.3%); 85 women (5.6%) were infected by a low-risk HPV and 253 with a high-risk HPV (16.7%).

The data in Table 1 represent the frequency of HPV infection observed in our 1518 women according to their age. There was a peak of infection in the third decade (31.1% of women) with a decrease after 60 years. There was also a significant prevalence of HPV infection in women who had a history of low- and/or high-grade lesions in a previous cervical smear or biopsy within the 2 years before examination. Twenty-eight (37.8%) of the 74 women with a history of lesions were infected with HPV versus 310 (21.5%) of 1444 women without medical history of SIL ($P < 0.001$).

### HPV detection and cytological and histological diagnoses

These results are summarized in Table 2. Overall, 1495 (90.8%) of the 1647 cervical samples included metaplastic and/or endocervical cells. There was a significant increase in the prevalence of HPV detection, especially high-risk HPV, related to the severity of the lesions ($P < 0.001$).

Twenty-five women had a smear evocative of a high-grade lesion at the first examination with a high-risk HPV (100%). This diagnosis was confirmed by the biopsy in these 25 patients. Fifty-six women had a smear evocative of low-grade lesions at the first examination with 43 high-risk HPV (76.8%) and four low-risk HPV (7.1%). The final histological diagnosis was a low-grade lesion in 42 cases and a high-grade lesion in two women. Twelve women did not present any cytological and histological lesion at the second control 40–120 days later. The absence of lesions was associated with the regression of HPV infection in seven of these cases (six high-grade and one low-grade) without any treatment and two persistent high-risk HPV infection.

Twenty-three women (1.5%) presented ASCUS. High-risk HPV DNA was present in 11 cases (47.8%). At the second control, one ASCUS corresponded to a high-grade lesion, five to a low-grade lesion and all these lesions were associated with high-grade HPV. The other 17 women did not present any detectable lesion at the colposcopy and/or the biopsy, but there was a persistent high-risk HPV infection in one case. At the third control, a high-grade lesion was detected in this woman.

In the remaining population of 1414 women with cervical smears within normal limits, high-risk HPV DNA was detected in 168 women (11.9%) and low-risk HPV in 85 cases (6.0%). In this population, 61 women with initial HPV infection had a follow-up: 42 with a high-risk HPV and 19 with a low-risk HPV. A persistent high-risk HPV infection at the second examination was observed in 26 out of 42 women and was associated with the detection of a high-grade lesion in five patients, 3–15 months after the first entry, and the detection of a low-grade lesion in five other women 4–10 months after the first examination. Three women with a high-risk HPV infection at the first examination had a low-risk HPV infection at the

*© 1999 Cancer Research Campaign*

second examination. A persistent low-risk HPV infection at the second examination was observed in eight out of 19 women and was associated with the detection of three low-grade lesions, within 4–12 months. Moreover, five other women of this group were secondly infected with high-risk HPV and developed three low-grade lesions within 6–14 months.

Thus in our series, 34 patients had a final diagnosis of high-grade lesion at the biopsy. All these women (100%) had previous scrapes positive for high-risk HPV. Earlier classic cytological screening revealed the absence of lesion in five cases (14.7%), the presence of ASCUS in two cases (5.9%) and cytological abnormalities evocative of low-grade lesion in two cases (5.9%) and of high-grade lesion in 25 cases (73.5%). In the 58 low-grade lesions, high-risk HPV were detected in 47 women (81.0%) and low-risk HPV in seven cases (12.1%).

### Pilot study: HPV testing on cone biopsy samples

On the 117 cone biopsies, the cervical scrapes performed on fresh tissue revealed the presence of high-risk HPV in 89 cases (76.1%). Seven of 20 inflammatory lesions (35.0%), seven of ten low-grade lesions (70.0%), 68 of 70 high-grade lesions (97.1%) and the seven invasive carcinomas (100%) were positive. The entire cone biopsy samples were histologically examined and so the final diagnosis was definitive. The absence of SIL in the 20 inflammatory lesions may be explained by excision-treatment with the biopsy or a spontaneous regression of the lesion.

### Evaluation of the viral load as a predictive parameter

Considering the viral load appreciated by the HC-II test, women with high-grade lesions had significantly higher signal strengths for high-risk HPVs than the rest of the population (Kruskal–Wallis test; $P < 10^{-8}$). In the same way, there was a significant difference in the viral load at the first examination for the 13 women with normal smears and a regressive high-risk HPV infection (mean signal strength of 74 units) and the 26 women with normal smears and a persisting high-risk HPV infection (mean signal strength of 190 units) especially when this infection was associated with the apparition of cervical lesions (mean signal strength of 320 units) ($P < 0.01$).

With a view to clinical application, we chose to test the sensitivity and specificity of the high-risk HPV DNA level for the detection of histologically proven high-grade lesions on our cone biopsy samples showing high-grade SIL and in samples obtained from 25 women without any CIN at the biopsy. The results obtained are summarized in Table 3.

Table 3 represents the respective sensitivity, specificity and positive predictive values of the various methods used for the detection of a high-grade SIL histologically proven.

### DISCUSSION

This work using the HC-II assay on a series of 1518 unselected women attending routine cytological screening clearly underlines the significant prevalence and/or persistence of HPV infection in women presenting precancerous and malignant cervical lesions. Also, there is a significantly increased prevalence of HPV detection, especially high-risk HPV, related to the severity of the lesion. Moreover, the prevalence of HPV infection is higher in women with a history of low- and/or high-grade lesions in a previous cervical smear or biopsy. HPV DNA has been detected in 400 (24.3%) of 1647 scrapes, with 296 (18.0%) containing a high-risk HPV. High-risk HPV was detected in all 34 cases presenting a high-grade lesion confirmed by biopsy, and in 68 (97.1%) of the 70 cone biopsy samples showing a high-grade SIL or an invasive carcinoma. Thus, the overall sensitivity of HC-II in detecting high-grade SILs and cervical cancers is 98.1%, and the specificity is 85.2% in cervical scrapes. The sensitivity of HC-II is quite similar to that of PCR using ‘consensus’ primers in previous studies (Van den Brule et al, 1991; Bosch et al, 1995). These data confirm our preliminary results comparing the respective sensitivities of HC-I, HC-II and PCR on 44 cone biopsy samples (Clavel et al, 1998*b*). Besides, in our experience, classic cytological screening gave positive results in 85.3% of the 34 high-grade lesions proven by the biopsy and its specificity was 94.9%. Thus even if it is less specific, HC-II represents a more sensitive test than classic cytology for the detection of high-grade cervical lesions.

In another way, we found 168 women (11.9%) infected with high-risk HPV positivities in a population of 1414 women with smears without any cytological abnormalities. The prevalence of HPV infection in our population is higher than that previously reported by other authors with the use of PCR (Walboomers et al, 1994; Rozendaal et al, 1996) and may be explained by our hospital recruitment with a subpopulation of women at high-risk. However, Rozendaal et al (1996) emphasized that the women with normal smears and high-risk HPV genotypes were 116 times more at risk of developing high-grade SIL, in contrast to women without high-risk HPV. Nevertheless the significance of such positivities and their predictive value are not totally resolved at the present time. Many HPV infections are known to regress spontaneously. In another way, in the literature, the persistence of high-risk HPV is significantly associated with progressive disease (Ho et al, 1995; Remmink et al, 1995). Our preliminary results on the follow-up of 61 women with HPV infection and smears within normal limits show that persistent HPV infection at a second examination in 42 cases was associated with the discovery of low-grade lesions in 11 cases and high-grade lesions in five cases within 3–15 months. The different modalities of collection of the samples may explain false-negative results of the cytology at the first entry. Cytology samples collected with Ayre’s spatula remove cells from the ectocervix and part of the transformation zone, whereas the cytobrush predominantly samples the endocervical canal as well as higher parts of the transformation zone. So these two different methods for collecting provide complementary data. Another source of cytological false-negative results is the rapid development of the lesions. Whatever the explanation of these apparently discrepant results, a more intensive cytological screening with HPV testing and clinical management with colposcopy should be offered in such high-risk HPV positive women without cytologic abnormalities, with an algorithm of 6 months to 1 year, to confirm the persistence of HPV infection and/or the occurrence of SIL.

Cuzick et al (1994) showed that in women with cytological abnormalities, HPV positivity at a high level detected by a semi-quantitative PCR was strongly related to high-grade CIN. Ho et al (1995) have also suggested that SIL with a high viral load are more likely to persist than those with a low level of HPV DNA. Thus, a high viral load may be considered as a risk factor and preferentially observed in potentially evolutive and in high-grade lesions. This parameter could be semi-quantitatively evaluated by the relative

© 1999 Cancer Research Campaign light unit values provided by the HC-II assay. In our previous study, the quantitative approach provided by the HC-I assay for the assessment of the viral load could not clearly distinguish between cases with or without high-grade lesions. Our present results with HC-II show that there is a highly significant correlation between a high level of high-risk HPV DNA and the severity of lesions. Moreover, a high viral load at a first examination is significantly associated with the persistence of viral infection or the apparition of a high-grade lesion in our series of 42 women followed up with a normal smear and a high-risk HPV infection. But, when this parameter is basically applied to differentiate patients with histologically proven high-grade SIL from women without any detectable lesions, it does not provide reliable sensitivity and specificity. Indeed, for a cervical sample/positive control ratio > 200, indicating a high viral load, the sensitivity is only 46.7% and the specificity 85.7%. However, we cannot exclude the possibility that women without cytological abnormal lesions, but with a high level of oncogenic HPV DNA, may represent a population at risk for the development of high-grade SIL or patients with an undetected SIL. Besides one major bias of this parameter evaluation remains the cell number in the cervical swabs which is not quantitated and can vary from one sample to another. In our experience at the present time, high-risk HPV DNA quantitation by HC-II cannot be considered a relevant test to be used in practice for the detection of high-grade lesions.

In conclusion, HPV detection with the HC-II assay represents a convenient and easy test for routine use. In our experience, its sensitivity for the detection of high-grade cervical lesions is higher than classic cytological screening, 98.1% versus 85.3% for cytology. This high sensitivity makes this assay highly recommendable for routine screening of high-grade lesions, in association with cytological examination. With the combination of the two methods, high-grade lesions would not be missed. The prognostic significance of a high level of high-risk HPV DNA detected with this assay has to be evaluated with repeat tests and follow-up of a large cohort of women. In a recent paper, Wright et al (1998) recommended the use of this test for older women (> 30 years old) and women with ASCUS. This would indeed improve the positive predictive value of the test. In our study, the application of this schedule of screening would increase the positive predictive value from 13.4% to 18.8%. However, considering the increasing occurrence of high-grade SIL in women under this age (nine cases out of 34 in our series of high-grade SIL), we propose to extend this indication to all women. At the present time, the cost of a HC-II test is about UK£25. Thus this proposition has to be evaluated in terms of cost-benefits in screening programmes.

### ACKNOWLEDGEMENTS

This work was supported by a grant from the ARC (Association de Recherche sur le Cancer), the ARERS, the CHU of Reims, the Ligue Nationale contre le Cancer (Comite's Marne-Aisne-Aube) and by the Lions Club of Soissons. We thank all the women who participated in this study, and Mrs Chantal Heller and Kathleen Thomas for their technical help.

### REFERENCES

Bosch FX, Manos MM, Munoz N, Sherman M, Jansen AM, Peto J, Schiffman MH, Moreno V, Kurman R, Shah KV and International Biological Study in Cervical Cancer Study Group (1995) Prevalence of human papillomavirus in cervical cancer: a worldwide perspective. *J Natl Cancer Inst* **87**: 796–802

Clavel C, Bory JP, Rihet S, Masure M, Duval-Binninger I, Putaud I, Lorenzato M, Quereux C and Birembaut P (1998*a*) Comparative analysis of the human papillomavirus detection by Hybrid Capture assay and routine cytology screening to detect high grade cervical lesions. *Int J Cancer* **75**: 525–528

Clavel C, Masure M, Putaud I, Thomas K, Lorenzato M, Bory JP, Gabriel R, Quereux C and Birembaut P (1998*b*) Hybrid Capture II, a new sensitive test for human papillomavirus detection. Comparison with Hybrid Capture I and PCR results in cervical lesions. *J Clin Pathol* **51**: 737–740

Cole HM (1993) Human papillomavirus DNA testing in the management of cervical neoplasia. *JAMA* **270**: 2975–2981

Cox JT, Lorincz AT, Schiffman MH, Sherman ME, Cullen A and Kurman RJ (1995) Human papillomavirus testing by hybrid capture appears to be useful in triaging women with a cytologic diagnosis of atypical squamous cells of undetermined significance. *Am J Obstet Gynecol* **172**: 946–954.

Cuzick J, Terry G, Ho L, Hollingworth T and Anderson M (1994) Human papillomavirus DNA in abnormal smears as a predictor of high-grade cervical intraepithelial neoplasia. *Brit J Cancer* **69**: 167–171

Farthing A, Masterson P, Mason WP and Vousden KH (1995) Human papillomavirus detection by hybrid capture and its possible clinical use. *J Clin Pathol* **47**: 649–652

Gaarenstroom KN, Melkert P, Walboomers JMM, Van den Brule AJC, Van Bommel PFJ, Meijer CJLM, Voorhorst FJ, Kenemans P and Helmerhorst TJM (1994) Human papillomavirus DNA genotypes: prognostic factors for progression of cervical intraepithelial neoplasia. *Int J Gynecol Cancer* **4**: 73–78

Hatch KD, Schneider A and Addel-Nour MW (1995) An evaluation of human papillomavirus testing for intermediate- and high-risk types as triage before colposcopy. *Am J Obstet Gynecol* **172**: 1150–1157

Ho GYF, Burk RD, Klein S, Kadish AS, Chang CJ, Palan P, Basu J, Tachzy R, Lewis R and Romney S (1995) Persistent genital human papillomavirus infection as a risk factor for persistent cervical dysplasia. *J Natl Cancer Inst* **187**: 1365–1371

Kaufman RH, Adam E, Icenogle J, Lawson H, Lee N, Reeves KO, Irwin J, Simon T, Press M, Uhler R, Entman C and Reeves WC (1997) Relevance of human papillomavirus screening in management of cervical intraepithelial neoplasia. *Am J Obstet Gynecol* **176**: 87–92

Koutsky LA, Holmes KK, Critchlow CW, Stevens CE, Paavonen J, Beckmann AM, Derouen TA, Galloway DA, Vernon D and Kiviat NB (1992) A cohort study of the risk of cervical intraepithelial neoplasia grade 2 or 3 in relation to papillomavirus infection. *N Engl J Med* **327**: 1272–1278

Lorincz AT (1996) Hybrid Capture method for detection of human papillomavirus DNA in clinical specimens. *Papillomavirus Rep* **7**: 1–5

Lorincz AT, Reid R, Jenson AB, Greenberg MD, Lancaster W and Kurman RJ (1992) Human papillomavirus infection of the cervix: relative risk association of 15 common anogenital types. *Obstet Gynecol* **79**: 328–337

Remmink AJ, Walboomers JMM, Helmerhorst TJM, Voorhorst FJ, Rozendal L, Risse EKJ, Meijer CJLM and Kenemans P (1995) The presence of persistent high-risk HPV genotypes in dysplastic cervical lesions is associated with progressive disease: natural history up to 36 months. *Int J Cancer* **61**: 306–311

Richart RM and Wright TC (1993) Controversies in the management of low grade cervical intraepithelial neoplasia. *Cancer* **71**: 1413–1421

Rozendaal L, Walboomers JMM, Van Der Linden JC, Voorhorst FJ, Kenemans P, Helmerhorst TJM, Van Ballegooijen M and Meijer CJLM (1996) PCR-based high-risk HPV test in cervical cancer screening gives objective risk assessment of women with cytomorphologically normal cervical smears. *Int J Cancer* **68**: 766–769

Schiffman MH, Kiviat NB, Burk RD, Shah KV, Daniel RW, Lewis R, Kuypers J, Manos MM, Scott DR, Sherman ME, Kurman RJ, Stoler MH, Glass AG, Rush BB, Mielzinska I and Lorincz AT (1995) Accuracy and interlaboratory reliability of human papillomavirus DNA testing by Hybrid Capture. *J Clin Microbiol* **33**: 545–550.

Sun XW, Ferenczy A, Johnson D, Koulos JP, Jungu O, Richart RM and Wright TC Jr (1995) Evaluation of the Hybrid Capture human papillomavirus deoxyribonucleic acid detection test. *Am J Obstet Gynecol* **173**: 1432–1437

Van Den Brule AJC, Walboomers JMM, Du Maine M, Kenemans P and Meijer CJLM (1991) Difference in prevalence of human papillomavirus genotypes in cytomorphologically normal smears is associated with a history of cervical intraepithelial neoplasia. *Int J Cancer* **48**: 404–408

Walboomers JMM, De Roda Husman AM, Snijders PJF, Stel HV, Risse EKJ, Helmelhorst TJM, Voorhorst FJ and Meijer CJLM (1994) Detection of genital human papillomavirus infections. Critical review of methods and prevalence studies in relation to cervical cancer. In: *Human Papillomavirus and Cervical Cancer. Biology and Immunology*, Stern PL and Stanley MA (eds), pp. 41–71. Oxford University Press Oxford:

 *© 1999 Cancer Research Campaign*

Wright TC Jr, Lorincz AT, Ferris DG, Richart RM, Ferenczy A, Mielsynska I and Borgatta L (1998) Reflex human papillomavirus deoxyribonucleic acid testing in women with abnormal Papanicolaou smears. *Am J Obstet Gynecol* **178**: 962–966

Zur Hausen H (1994) Molecular pathogenesis of cancer of the cervix and its causation by specific human papillomavirus types. *Curr Top Microbiol Immunol* **186**: 131–156

*© 1999 Cancer Research Campaign*
